# Larvicidal, Histopathological Efficacy of *Penicillium daleae* against Larvae of *Culex quinquefasciatus* and *Aedes aegypti* Plus Biotoxicity on *Artemia nauplii* a Non-target Aquatic Organism

**DOI:** 10.3389/fphar.2017.00773

**Published:** 2017-10-27

**Authors:** C. Ragavendran, T. Mariappan, Devarajan Natarajan

**Affiliations:** ^1^Natural Drug Research Laboratory, Department of Biotechnology, School of Biosciences, Periyar University, Salem, India; ^2^ICMR-Centre for Research in Medical Entomology, Madurai, India

**Keywords:** *Penicillium daleae*, larvicidal, KX387370, phylogenetic tree, *Artemia nauplii*, bio-toxicity

## Abstract

Mosquitoes can transmit the terrible diseases to human beings. Soil-borne fungal products act as potential source for low-cost chemicals, used for developing eco-friendly control agents against mosquito-vector borne diseases. The prime aim of study was to check the larvicidal potential of fungus mycelia (by ethyl acetate solvent) extract from *Penicillium daleae* (KX387370) against *Culex quinquefasciatus* and *Aedes aegypti* and to test the toxicity of brine shrimp *Artemia nauplii*, by observing the physiological activity. The ethyl acetate extract of *P. daleae* mycelia (after 15 days) from Potato dextrose broth (PDB) medium revealed better result with least LC_50_ and LC_90_ values of I-IV instars larvae of *Cx. quinquefasciatus* (LC_50_ = 127.441, 129.087, 108.683, and 93.521; LC_90_ = 152.758, 158.169, 139.091, and 125.918 μg/ml) and *Ae. aegypti* (LC_50_ = 105.077, 83.943, 97.158, and 76.513; LC_90_ = 128.035, 106.869, 125.640, and 104.606 μg/ml) respectively. At higher concentration (1000 μg/ml) of extracts, mortality begins at 18 h of exposure and attained 100% mortality after 48 h exposure. Overall, the activity was depends on the dose and time of exposure to the extracts. The stereomicroscopic and histopathological analysis of *Ae. aegypti* and *Cx. quinquefasciatus* larvae treated with mycelium ethyl acetate extract showed complete disintegration of abdominal region, particularly the midgut and caeca, loss of cuticular parts and caudal hairs. Morphological characterization of the fungi was performed and taxonomically identified through 5.8s rDNA technique. The phylogenetic analysis of rDNA sequence was carried out to find out the taxonomic and the evolutionary sketch of isolate in relation to earlier described genus *Penicillium*. Behavior and swimming speed alteration was analyzed together with mortality. The results of the experiment indicates that swimming behavior recorder (SBR) is a appropriate tool to detect individual swimming speed of the *A. nauplii* organisms, since the values have been obtained in accordance with control monitored results showed the 2.75 mm s^-1^ and after 24 h treated found to be 0.72 mm s^-1^, respectively. The extract-exposed to *A. nauplii* showed changes in body structures, i.e., intestine enlargement, eye formation, outer shell malformations and loss of antennae. In the present study, we aimed to investigate the toxicity of the ethyl acetate extract of *P. daleae* on *A. nauplii* larvae by performing the mortality, behavior and alterations in swimming responses. This is the first time report on the larvicidal efficacy of *P. daleae* ethyl acetate extract against *Cx. quinquefasciatus* and *Ae. aegypti* larvae.

## Introduction

Mosquitoes borne diseases are responsible for most significant health problems that have pose considerable amount of social and economical impacts (James, 1992; Benserradj and Mihoubi, 2014). *Culex quinquefasciatus* (Say) (family Culicidae) is a major contributor for transmitting vector borne diseases like fevers and filariasis to human beings and animals. It can also spread St. Louis encephalitis and possibly the West Nile virus. The parasitic filarial worms *Wuchereria bancrofti* and *Brugia malayi* might cause lymphatic Filariasis (Soni and Prakash, 2010; Benelli, 2015a,b). More than 40 million peoples affected globally from Elephantiasis and are badly debilitated and disfigured from this disease (WHO, 2013).

In worldwide, yellow, chikungunya and dengue fever, transmitted by *Aedes aegypti* has currently become a significant community health problem (Warikoo et al., 2011). Dengue fever is caused by dengue virus, which is belongs to the genus *Flavivirus* (family Flaviviridae) and includes serotypes (Den-1, Den-2, Den-3, and Den-4). Those diseases were came-up in the middle of the 20th century and considered as a most important dreadful disease in several countries of tropical and subtropical regions (Vasilakis et al., 2007; Benelli, 2015b). Totally, 2.35 million people suffering from dengue was reported in America alone (as per 2013 data) of which 7,687 cases were rigorous (WHO, 2015). Recently, the infections have been raised due to an increased urbanization, trade, and travel. No effective drug or vaccine is available so far. But, the only solution is to prevent the disease-carrying mosquito from breeding and biting humans. Currently, the application of chemical based insecticides particularly organophosphates and insect growth regulators namely diflubenzuron, malathion, pyrethroid, and methoprene served as common agents for mosquito control (Conti et al., 2012). Frequent uses of these synthetic insecticides create an increase resistance among the mosquitoes and to cause adverse impacts on non-target organisms or humans (Severini et al., 1993). With this scenario, biological control especially fungal based insecticide has proven as better in controlling insect vectors.

In recent years, several attempts have been carried out to examine the bioefficacy of natural products against a variety of arthropod pests (Amer and Mehlhorn, 2006b; Govindarajan et al., 2011; Benelli et al., 2012a,b; Govindarajan and Sivakumar, 2012). Moreover, the researchers are searching for an alternative, more potent, cost effective and environment-friendly mosquito vector control agents from biological origin (Regnault-Roger et al., 2012; Pavela, 2015a,b). Different mosquito larvicidal bio-control agents have been isolated from various sources like plants, bacteria, fungi, and viruses that infect or kill insects by their potent secondary metabolites (Vyas et al., 2007). Fungi and fungus derived products are very highly toxic to mosquitoes, and reflect moderate toxicity to non-target organisms. Among fungi, entomopathogens are extremely important source of potential biological control agents (Berdy, 2005). Several authors have been reported mosquito bio-efficacy of the soil-borne fungal metabolites or mycelia extracts of *Aspergillus* sp., *Fusarium sporotrichoides* and *Penicillium verrucosum* (Maurya et al., 2011), *Aspergillus terreus* (Ragavendran and Natarajan, 2015). Previously, many extracellular secondary metabolites isolated from different fungi have been proved as potent larvicidal activity against targeted mosquitoes (Vijayan and Balaraman, 1991) viz *Metarhizium* sp. (Roberts, 1966, 1967), *Beauveria* sp. (Hamill et al., 1969), *Tolypocladium* sp. (Matha et al., 1988), and *F. oxysporum* (Peter et al., 1989; Prakash et al., 2010), *Lagenidium giganteum* (Vyas et al., 2006a,b) and *Chrysosporium* (Priyanka et al., 2001; Priyanka and Prakash, 2003), respectively.

*Penicillium* is one of the most important fungi (ascomycetes) in the field of drug production (Tiwari et al., 2011). *Penicillium* sp. are identified to produce more than 900 known bioactive compounds (Berdy, 2005). In addition, most essential pharmaceutical agents (penicillin and compactin), have been isolated from *Penicillium*. Many species of *Penicillium* sp. produces highly toxic mycotoxins (Kamel and Rashed, 2014). *P. daleae* is first isolated from conifers in Poland (Lam et al., 1994). The microbial insecticides play an important role in exhibiting better mosquito larval toxicity, safer to produce effect on non-target organism and environmental hazards (Misato, 1983).

*Artemia nauplii* (Zooplankton) constitutes a major relationship in the food chain and has significant role in aquaculture field (Radhika Rajasree et al., 2010) also called as an ecologically essential species (Mehmet et al., 2016). It consumes organic debris, micro and macro algae, bacteria and chemicals (Weber, 1993). Over the past few decades, the toxicity of *A. nauplii* has been tested with various bioactive or toxic compounds, pesticides, antimicrobial biocides expressed some behavioral responses (Sorgeloos et al., 1978; Venkateswara Rao et al., 2007; Garaventa et al., 2010; Alyuruk et al., 2013) and this species used as an excellent organism for eco-toxicological tests (Persoone and Wells, 1987; Garaventa et al., 2010). *A. nauplii* is used as a model organism for testing the toxicity of ethyl acetate in *P. daleae* according to the Organization for Economic Cooperation and Development testing guidelines (OECD) (OECD, 2004). This is the first time report on the larvicidal efficacy of *P. daleae* ethyl acetate extract against *Cx. quinquefasciatus* and *Ae. aegypti* larvae and check the toxicity of *A. nauplii* larvae by performing the mortality, behavior and alterations in swimming responses.

## Materials and Methods

### Soil Sample Collection

The soil sample was collected from forests area in Karumandurai hills, Salem District (latitude 78°20 and longitude 11°45). Sample were placed in sterile polyethylene bags and stored at 4°C for further study.

### Fungus Isolation

Soil fungus was isolated using the modified soil-dilution method (Warcup, 1950, 1955) and three different soil dilutions (viz., 10^-1^, 10^-2^, and 10^-3^) were employed for fungal isolation. The soil sample (1 g) was suspended in sterile distilled water (100 ml) using sterilized glass test tube. These suspensions were stirred for 20 min. To prepare 10-fold dilution series, 1.0 ml of the soil suspension was added to 9 ml of sterilized distilled water (10^-1^) and mixed well for 2 min for homogenization. In 10^-2^ dilution, 1.0 ml diluted soil suspension (10^-1^) was added to 9 ml of sterilized water. To make 10^-3^ dilution, 1.0 ml diluted suspension (10^-2^) was transferred to 9 ml of sterilized water and mixed well. The fungal isolation (from each soil dilution) was carried out by uniform spreading of 0.1 ml homogenized suspension (Phara and Kommedahl, 1954) on the surface of medium with the help of sterilized glass rod and incubated at 25 ± 2°C for 3–5 days. All the fungal isolates were purified by single spore isolation technique (Choi et al., 1999). Pure fungal cultures were used for identification and preservation.

### Culture Conditions

The isolated fungus was observed morphologically and cultured in Potato Dextrose Agar (PDA) and Sabouraud Dextrose Agar (SDA) medium containing sterile Petri dishes and incubated at 37°C for 7 days under dark (Incubator), based on the modified methods of Pitt (1979) and Domsch et al. (1980). The color names and codes were used to describe well grown colony (Kornerup and Wanscher, 1967).

### Morphological (Macroscopic and Microscopic) Characterization

Pure culture of isolated fungus were identified based on the macro (colony morphology, color and appearance of colony, and shape) and microscopic features (septation of mycelium, shape, size, diameter and texture of conidia) with the help of standard keys (Raper and Thom, 1949; Pitt, 1979; Domsch et al., 1980). Pure fungal isolates were observed under day light for culture appearance and further examination has been undertaken using needle mount preparation methods (Tuite, 1961; Barron, 1968; Crowley et al., 1969; Burnett, 1975). In addition, the microscopic study of isolated fungus (*Penicillium* sp.) was done by preparing a new clean slide mount with lacto-phenol cotton blue stain and the results were observed microscopically (Lobomed at 40×) (Klich and Pitt, 1992).

### Fungal Mycelium Preparation and DNA Isolation

Fungal mycelium was prepared from pure culture using 50 ml of PDB broth (100 ml conical flasks) and incubated at 25 ± 1°C (for 7 days). Mycelium (50 ml broth) was harvested by filtration using Whatman no.1 filter paper (1 cm in diameter) from pure *Penicillium* sp. were inoculated aseptically to cultivate in 100 ml liquid medium (PDB) (at 25°C for 7 days). The fungal mats were recovered from flasks and dried with filter paper. For extraction of total DNA from the mycelia and conidia of the isolates were done by the modified 2% CTAB method (Doyle, 1990; Rolhf, 1990). *P. daleae* mycelium (1 g) mat was grind to make fine powder in liquid nitrogen with help of pre-cooled clean pestle and mortar. Then, CTAB buffer (1 ml) was added immediately after crushing and mixed with grind material. The mixture was transferred to sterilized eppendorf tubes and placed in (pre-heated) heat shock at 65°C (for 30 min) and incubated with providing an intermittent shaking (15 min intervals). After incubation, the tubes were cooled for 3–4 min in an ice and centrifuged (Remi centrifuge) at 8,000 rpm (for 12 min). The supernatant was collected into newly labeled eppendorf tubes. Equal volume of chloroform-iso amyl alcohol (24:1 v/v) was added and gently agitated for 10 min to form an emulsion. Centrifugation was performed at 12,000 rpm for 10 min and the supernatant was transferred to sterile eppendorf tube. Then, 2/3 volume of chilled isopropanol and 100% ethanol was added to each eppendorf tube, mixed well and incubated at -20°C (for 1 h). After incubation, centrifugation was performed at 12,000 rpm for 15 min. At this stage, the DNA pellet was obtained and the supernatant was discarded. The pellet was washed with 70% ethanol to remove any residual salt and allowed to air dry. Finally, pellet was dissolved in 150 μl TE (Tris-EDTA) buffer. The DNA concentration purity was estimated by the measurement the optical density (OD) (at wavelength 260 and 280 nm) in spectrophotometer (Shimadzu U160A, Japan). DNA integrity was also checked by 0.8% agarose gel-electrophoresis. The purified genomic DNAs were further cleaned by additional RNase treatment and sequential washing steps were carried out with phenol/chloroform and chloroform to attain high quality DNA sample for sequencing. Pure DNAs were stored at -20°C for further investigation.

### Polymerase Chain Reaction

Primers chosen for amplification of DNA as Internally Transcribed Spacer (ITS) gene points of the 5.8S rDNA were ITS (5′ – GRAAGNAHADGTVGKAAYAWSG – 3′) and ITS (5′ – TCCTNCGYTKATKGVTADGH – 3′) (White et al., 1990). Amplification of the ITS gene region was performed in a 100 μl reaction volume and the protocol per reaction contained 1.0 μl of genomic DNA (100 ng), 1.0 μl (400 ng) Forward Primer, 1.0 μl (400 ng) Reverse Primer, 10 μl of Taq DNA Polymerase assay buffer, 1.0 μl Taq DNA Polymerase enzyme, 10 mM of each of the four dNTPs, 0–4% (of the final volume) stock dimethyl sulfoxide. The polymease chain reaction (PCR) conditions as follows: initial denaturation (at 95°C for 5 min), 35 cycles of denaturation (at 94°C for 30 s) annealing (at 50°C for 30 s), and elongation (at 72°C for 1 min 30 s) with final extension (at 72°C for 7 min), respectively.

### Agarose Gel Electrophoresis

Polymease chain reaction products were analyzed on 1.5% agarose gel. For making this gel, 1.5 g of agarose was weighed and poured into a 250 ml beaker. Then, 100 ml of 1X TBE (Tris-Boric Acid-EDTA) buffer was added to the beaker. To dissolve the agarose completely in the buffer, beaker was heated in a microwave (for approximately 2 min). After agarose was dissolved, the solution was allowed to cool down (60°C), added 5 μl of Ethidium bromide (EtBr) and mixed for visualization of bands. Luke warm gel was poured into the castner after that combs were fixed at their specific position. Gel was allowed to solidify (approximately 15 min). Gel tank was filled with 1X TBE buffer served as a running buffer. Then, the combs were removed and the solidified gel was placed in the tank. DNA sample (5 μl) from each tube was mixed with 2 μl loading dye and filled in gel wells. Gel electrophoresis (Electrophoretic gel System, EC 330, Thermo electron Corporation, United States) was performed at 80 v for about 30 min. Amplified products were visualized by placing the gel in UV documentation system (Bio-Rad, Italy).

### Sequence Analysis

The DNA was sequenced at the Chromous Biotech Pvt. Ltd. (Tamil Nadu, India), and the sequence was submitted to GenBank. Sequences results were compared to the GenBank data bases by BLAST analysis^1^. Results having entire species identification were done from NCBI Genbank database. Thereafter, the order of organisms were aligned with IN-5 using CLUSTAL W^2^ program. CLUSTALW (BioEdit) (Hall, 1999) used for multiple alignments of sequences. The nucleotide and deduced amino acid sequences homology was calculated by MegAlign (DNA Star, Inc., Madison, WI, United States). MEGA5 (Tamura et al., 2011), distance matrix, Neighbor-Joining (Saitou and Nei, 1987) and maximum parsimony methods (Tamura et al., 2004) were used for phylogenetic analysis (FigTree V1.3.1software) (Rambaut, 2009).

### Extract Preparation

The mass cultivation of isolated pure *P. daleae* (static condition) grown in Potato Dextrose broth (PDB) was kept under constant temperature of 20°C and 25 days old culture was used to preparation of ethyl acetate mycelium extract. At the end of growth period, the collected mycelia were extracted separately using solvent extraction method (Lin et al., 2000). The fresh and well grown mycelia of fungus was washed thrice with sterile distilled water for removal of adherent filtrate, medium and plotted between folds of sterilized Whatman filter paper (no. 1). The plotted mycelium was crushed by sterile surgical blade (in mortar), extracted with ethyl acetate to obtain intracellular metabolites. Both crushing and extraction was repeated for three times, left in separating funnel for 15 min to precipitate. The crude ethyl acetate extract was collected. Using a separating funnel, the filtrate of fungus was extracted in several times using ethyl acetate solvent and evaporated under vaccum pressure at 50°C until the complete dryness. The resulted semi-solid material was dissolved in 10% DMSO and kept for further investigation (Belofsky et al., 1998; Holler, 1999).

### *Culex quinquefasciatus* and *Aedes aegypti* Rearing

Egg rafts of *Cx. quinquefasciatus* and *Ae. aegypti* were provided by the Centre for Research in Medical Entomology (CRME) Madurai (Tamil Nadu, India). Freshly collected eggs were transported to laboratory conditions [27 ± 2°C, 75–85% R.H., 14:10 (L:D) photoperiod] and placed in plastic containers (16 cm × 12 cm × 4 cm) having distilled water (1000 ml) and kept for hatching. The mixture of dog biscuits and yeast (3:1 ratio) was administered as food supplements for larvae. The matured larvae and pupae were collected and transferred to plastic containers with 500 ml of water and used for an experimental purpose (Senthil-Nathan et al., 2006).

### Larvicidal Activity

Larvicidal activity of the ethyl acetate extract of *P. daleae* mycelium was evaluated as per the modified WHO protocol (World Health Organization, 2005). From the stock solution of the mycelial ethyl acetate extract (1 mg/ml), different concentrations of tested samples were prepared and tested (300, 500, 800, and 1000 μg/ml) as per the modified procedure of Benelli (2015a). The I-IV instar larvae (each 20 No’s) were introduced in 100-ml glass beaker containing 99 ml of dechlorinated water, and add 1 ml of ethyl acetate extract (known concentration). Five replicates were maintained (100 larvae) in each concentration. The mortality of larvae observed at 48 h after exposure. No food was provided to the larvae during the experimental time. A set of control groups (10% DMSO and distilled water) along with five replicates for individual concentration were maintained in each test. The lethal concentration level (LC_50_ and LC_90_) was calculated by probit analysis (Finney, 1971). The observed/corrected mortality of five replicates and combined for each concentration, was calculated as per Abbott’s formula (Abbott, 1925).

$$\begin{array}{c} \mathrm{Corrected}mortality =\frac{\begin{array}{l} \mathrm{Obtained}\mathrm{mortality}(\mathrm{in}\mathrm{treatment})-Obtained\mathrm{mortality}(\mathrm{in}\mathrm{control}) \end{array}}{\begin{array}{l} 100-Control(\mathrm{mortality}) \end{array}}\times 100 \end{array}$$

### Histopathological and Stereomicroscopic Analysis

Thin section of *Ae. aegypti* and *Cx. quinquefasciatus* larval tissues treated with *P. daleae* mycelial ethyl acetate extract were prepared (8 μm thickness) using microtome. Using a clean glass slide, the sectioned tissues were mounted and stained using hematoxylin and eosin stain. The effects of larval toxicity of extract were observed under bright field light microscope at 40×. The extract accumulation of tissue and its alterations were seen under a stereomicroscope (Banumathi et al., 2017).

### Cultivation of Model Organism

*Artemia nauplii* cysts were hatched in seawater (30% m/v) by dissolving appropriate amount of Bay of Bengal Ocean salt in deionized water and stirred for 36 h under proper aeration, and it was filtered through 30-μm Millipore filter. The hatching of *A. nauplii* was done as per the protocol of Persoone et al. (1989). Briefly, encysted *A. nauplii* was first hydrated in distilled water at 4°C for 12 h and then washed to separate the floating cysts from the conical flask. The sinking cysts were collected using a Buchner funnel and washed with cold deionized water. Approximately, 2 g of the pre-cleaned cysts were added in 1.0 L seawater in a conical flask with 30 ± 3°C temperature (A1500 lx daylight supplied regularly by a fluorescent lamp) and the aeration was maintained (small line extending to the bottom of the hatching device from the air pump of an aquarium). After 36 h, the hatching of *A. nauplii* was noticed.

### Counting of Hatched *Artemia*

The counting of hatched *Artemia* was performed as per the modified method of Sorgeloos (1980).

The hatched *A. nauplii* containing sample (100 ml) was taken in a clean glass beaker. From this stock, 1 ml of sample diluted with 100 ml of sea water. 0.1 ml of diluted sample was poured in sterile petri dishes and the number of *A. nauplii* was counted by visual observation.

### Preparation of *P. daleae* Mycelium Ethyl Acetate Extract

The suspension of extract was prepared in between 200 and 300 μg/ml to record considerable activity. About 20% (m/v) stock suspension was prepared by dissolving known amount of mycelial ethyl acetate extract in 10% DMSO. The prepared sample was vortexed for 60 s, and it was sonicated about 5 min, for maximum dissolution. Desired amount of the stock suspension were transferred immediately into the exposure beaker containing *A. nauplii*.

### Exposure Studies

Acute toxicity exposure of sample was conducted on *A. nauplii* (for 36 h), based on the guidelines of Organization for Economic Cooperation and Development (OECD, 2004). Three different test concentrations (50, 200, and 300 μg/ml) of *P. daleae* mycelial ethyl acetate extract was given to *Artemia* larvae and also maintain a control group (avoid the test sample). The experiments were done in triplicates using clean glass beaker (100 ml). Exposures were conducted in 100 ml seawater for *A. nauplii* and proper aeration was provided (line extending to the bottom of the conical flask to prevent the deposition of extract from suspension during the exposure times). Light regime of 16:8 h light/dark and temperature was set at 25 ± 2°C. The pH of the solution was measured before and after completion of experiments. No food was given during the course of the experiment (Sivagnaname and Kalyanasundaram, 2004).

### Behavior and Swimming Speed Alteration Test

Swimming speed alteration tests of sample was performed by the modified method of Garaventa et al. (2010). In briefly, after 6 h exposure of the *A. nauplii* larvae to the ethyl acetate extract of *P. daleae*, the swimming speed was recorded using a video camera with a macro objective. The inner part of apparatus was covered with black color (30 cm × 30 cm × 60 cm) to exclude external sources of fluorescent light, and the recording chamber was monitored under infrared light. The *A. nauplii* larvae were kept in dark condition for 5 min before it was recorded. The swimming behavior was digitally recorded for about 4 s at 25 frames/s, and the images were analyzed using advanced image processing software to reconstruct the individual tracks, with the measurement of the swimming speeds (mm/s) for each *A. nauplii* larva in each sample (20–25 larvae/concentration). The data are referred to as the swimming inhibition, following normalization to the swimming speed of the controls (S, mean swimming speed), where

Inhibition (%) = [(S Treated - S Control)/S Control × 100].

### Data Analysis

The probit analyses of obtained results were done for LC_50_, LC_90_, 95% confidence limits and chi-square values. The Statistical Package for the Social Sciences (SPSS) 20.0 software used for analyses the derived results and the significance (*p* < 0.05) level also measured.

## Results

Macroscopic features of isolated *P. daleae* (Petri-dish containing PDA media) showed rapid growth, 25 ∼ 35 mm diameter, plane or lightly radially sulcate, velutinous, mycelium white, conidiogenesis was very light to dark green color, of an granular powdery colony (front) and pale yellow colony (backside). Whereas, on Sabouraud’s Dextrose Agar (SDA), this species showed moderate growth, greenish orange color in granular form and the colony produce yellow orange in color (backside), absence of exudate or abundant in small drops and it produces yellow to reddish brown pigment and reverse side shown yellow brown color (Zaleski, 1927).

**FIGURE 1:**
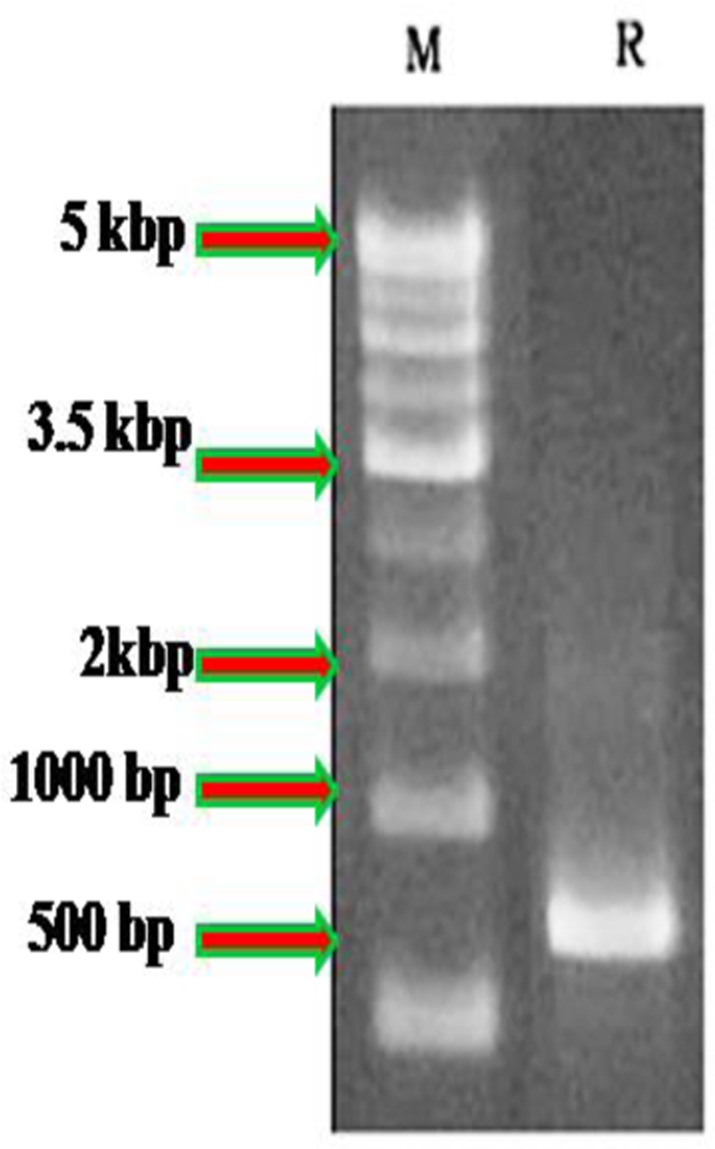
Molecular analysis of *Penicillium daleae* using ITS1-5.8S–ITS2 rRNA gene compared with 500 bp marker. M, ladder; R, *P. daleae*.

**FIGURE 2:**
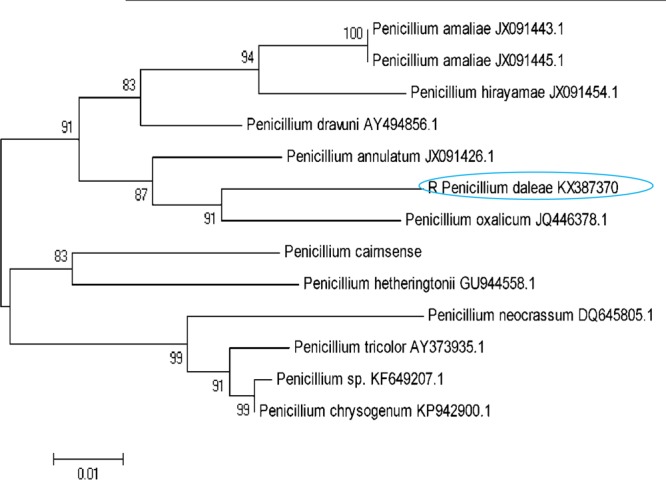
A neighbor-joining tree of the *Penicillium* species relative to the *P. daleae* isolate based on phylogenetic analysis of the nuclear ribosomal ITS1-5.8S-ITS2 region sequences. Bootstrap values of 1000 analyses are shown at the branching point. The type strain of the species and accession numbers are indicated as “T” and in parentheses, respectively (Tamura et al., 2011).

The isolated DNA sample was amplified in optimized condition using the universal primers and the rDNA fragments were shown in **Figure 1**. The 5.8 S rDNA gene is the generally used marker for inferring the phylogenetic relationship among fungal species due to its well conserved nature (Woese et al., 1985). The obtained bands were located in the expected ITS region (ranging from 400 to >1000 bp), with approximately 600 and 650 bp, respectively. The obtained rDNA sequence of the isolated coding 5.8S rDNA gene was deposited in GenBank database (NCBI) provided the Accession No KX387370. Subsequently, based on 5.8S rDNA sequence analysis, the strain showed 99% homology/similarity with an already reported *Penicillium* sp. sequences. On the basis of all (morphological, physiological, and molecular) characteristics of the isolates identified as *P. daleae*. The evolutionary history of the isolates was inferred by the Neighbor-Joining tree method. The optimal tree was constructed with the sum of branch lengths (=0.33604926) (**Figure 2**). The percentage of replicate trees are calculated on the basis of associated taxa clustered together in the bootstrap test (1000 replicates) has been revealed next to the branches. Tamura et al. (2011) method was used for computing and plotted the evolutionary relationships and units number of bases per substitution site. The phylogenetic tree analyses involved 13 different fungal nucleotide sequences. All positions with <95% location coverage were removed and less than 5% of arrangement gaps, lost data and unclear nucleotide bases were approved at 418 positions during phylogenetic construction.

In laboratory mosquito bio-assays, the toxicity of mycelia ethyl acetate extract of *P. daleae* against larval instars (I–IV) of *Cx. quinquefasciatus* resulted the least LC_50_ and LC_90_ values: (LC_50_ = 127.441, 129.087, 108.683, and 93.521; LC_90_ = 152.758, 158.169, 139.091, and 125.918 μg/ml) and *Ae. aegypti* (LC_50_ = 105.077, 83.943, and 76.513; LC_90_ = 128.035, 106.869, 125.640, and 104.606 μg/ml), respectively (**Table 1**). After treatment of *P. daleae* metabolites, significant reduction of larvae against *Ae. aegypti* was recorded. The mortality rate of *Cx. quinquefasciatus* was slower, but larvae suffered with severe deformities. The sub lethal effects on fourth instars larval were linked with decreased survival of the early instars. Third instar larvae were highly susceptible at the optimized concentrations. At 1000 μg/ml concentration of *P. daleae*, metabolite showed strong activity against *Ae. aegypti* larvae and the mortality rate was higher than *Culex*.

**Table 1:** Larvicidal activity of *Penicillium daleae* fungal mycelium (ethyl acetate) extract against *Culex quinquefasciatus* and *Aedes aegypti* (after 48 h exposure).

Mosquito species	Larvae stages	Concentrations (μg/ml)	LC_50_ (LCL-UCL) (μg/ml)	LC_90_ (LCL-UCL) (μg/ml)	χ^2^ (*df* = 10)
*Cx. quinquefasciatus*	I	300	127.441	152.758	4.891
		500	(82.083–167.917)	(103.945–194.810)	
		800			
		1000			
	II	300	129.087	158.169	4.848
		500	(80.712–172.917)	(105.296–204.163)	
		800			
		1000			
	III	300	108.683	139.091	3.603
		500	(57.446–157.269)	(80.517–191.388)	
		800			
		1000			
	IV	300	93.521	125.918	4.275
		500	(39.045–147.987)	(60.255–186.029)	
		800			
		1000			
*Ae. aegypti*	I	300	105.077	128.035	3.470
		500	(59.933–146.662)	(78.223–171.873)	
		800			
		1000			
	II	300	83.943	106.869	2.702
		500	(39.484–128.153)	(55.327–154.927)	
		800			
		1000			
	III	300	97.158	125.640	4.469
		500	(47.549–145.486)	(67.988–178.171)	
		800			
		1000			
	IV	300	76.513	104.606	5.792
		500	(27.317–128.882)	(43.593–163.406)	
		800			
		1000			

*Control (deionized water) – nil mortality. LC_*50*_, lethal concentration that kills 50% of the exposed larvae; LC_*90*_, lethal concentration that kills 90% of the exposed larvae, LCL, lower confidence limit; UCL = upper confidence limit; *df*, degree of freedom; χ^*2*^, chi-square values are significant at *P <* 0.05 level. Mean value of five replicates.*

**FIGURE 3:**
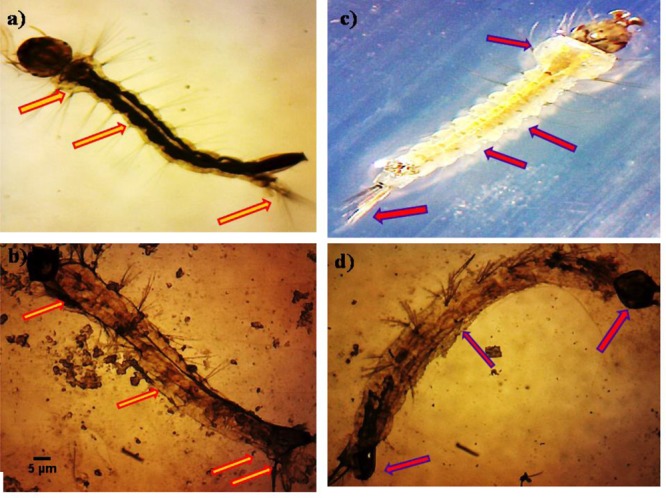
Stereo microscopic study of third instar **(a,c)** control larvae of *Aedes aegypti* and *Culex quinquefasciatus* of midgut, thorax, and anal gill parts. **(b,d)** *Ae. aegypti* and *Cx. quinquefasciatus* treated with *P. daleae* mycelium extract at 1000 μg/ml concentration and extract treatment induced toxic effects on many regions of the body including thorax, abdomen, anal gills, (red and violet arrows) indicates loss of external hairs, crumbled epithelial layer of the outer cuticle and shrinkage of the larvae, respectively.

The untreated *Ae. aegypti* and *Cx. quinquefasciatus* (3rd instar larvae) showed normal appearance with having well-developed and well-known eye, head, thorax, and abdomen segments of whole body (**Figures 3a,b**). The *P. daleae* mycelium ethyl acetate extract treated with larvae of *Ae. aegypti* resulted pellitorine produce dark color, thorax and abdominal segments (1st–5th) are predominately damaged. The damaged internal gastric caeca and dark black spots in the thorax and anal gills were observed (**Figure 3c**). Stereomicroscopic observation on 3rd instar larvae of *Cx. quinquefasciatus* showed crumbled epithelial layer of the outer cuticle and lack of external hairs (upper and lower head hairs, lateral, antenna, and caudal hairs) after treated with *P. daleae* mycelium extract (**Figure 3d**).

**FIGURE 4:**
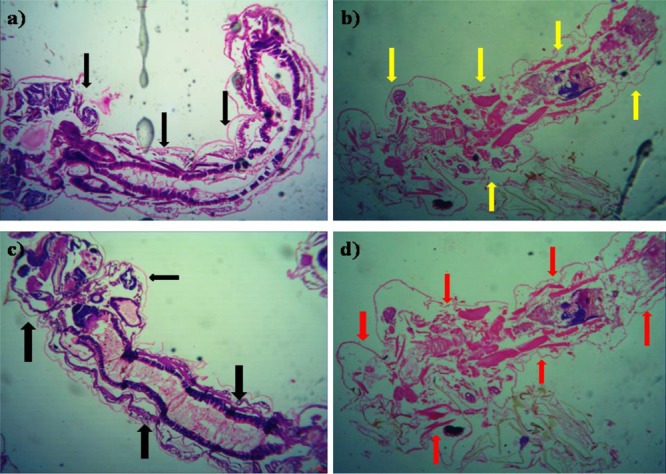
Histopathological study on treated third instar larvae of *Ae. aegypti* and *Cx. quinquefasciatus*: Control **(a–d)** treated with the *P. daleae* mycelium ethyl acetate extract, *Black* arrow represents control larvae structures, *yellow* and *red* arrow indicates the disordered and broken epithelial cell layer, complete breakup of mid-gut and caeca and collapsed larval structure, respectively.

The histopathological study of *P. daleae* ethyl acetate extract treated with *Ae. aegypti* and *Cx. quinquefasciatus* larvae showed collapse and brokened epithelial cell layers. Whereas, target mosquitoes showed an entire damage in the mid-gut and caeca areas and finally, larval structure was fully collapsed (**Figure 4**). Moreover, a huge shrinkage was observed in the abdominal region of *Ae. aegypti* and *Cx. quinquefasciatus* larvae treated with mycelial ethyl acetate extract. To the best of our knowledge, reports on histopathological changes triggered by fungal based metabolites are not available so far.

In control *A. nauplii* show an average speed of 2.75 mm s^-1^ after cultured in the testing wells (6 and 48 h). After 24 h treated with ethyl extract of *P. daleae* the swimming speed values of were found to be 0.72 mm s^-1^, respectively. Behavioral changes in the swimming activity and the position of the *A. nauplii* were noticed in the 6 h incubations with *P. daleae* mycelium extract in stationary phase. After 12 h exposure to toxic cells, either in exponential phase, individual *A. nauplii* have shown changes in swimming activity and in their position on the water column. The results of the swimming speed alterations are showed more than 50% mortality was observed in the larvae exposed to three different concentrations of the *P. daleae* mycelium ethyl acetate extract. Although there was maximum percentage of swimming alteration observed at 50 μg/ml concentration and swimming was notably inhibited in larvae exposed at 300 μg/ml.

**FIGURE 5:**
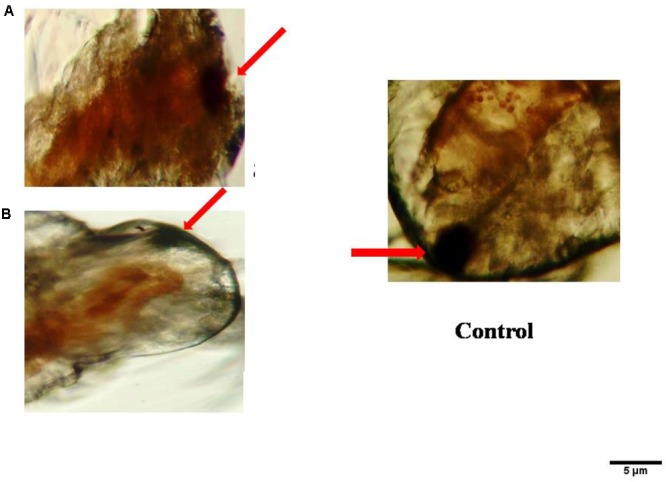
Visible anomalies in *Artemia nauplii* subjected to different concentrations of *P. daleae* mycelium ethyl acetate extract. Control *A. nauplii* not exposed to any extract. While the normal eye is round and black (control), extract exposed nauplii had changes in the eyeball shape **(A)**, loss of eye color, and fading of eyes **(B)**. The *A. nauplii* were observed periodically under light microscope (Labomed at 40x).

**FIGURE 6:**
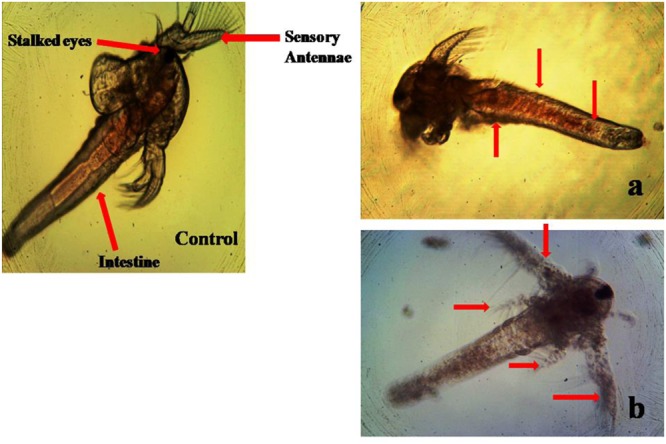
Morphological changes of *A. nauplii* exposed to *P. daleae* mycelium ethyl acetate extract. *A. nauplii* not exposed to extract (control), intestinal enlargement and outer shell anomalies **(a)** and loss of antennae and deformation of antennae **(b)**.

No death *A. nauplii* was noticed during the toxicity test in the control groups. The toxicity of mycelial ethyl acetate extract to *A. nauplii* increased with rising extract concentrations and exposure times. Various concentrations were treated and LC_50_ values calculated that killed *A. nauplii* (50% of *nauplii*) (**Table 2**). The control group of *A. nauplii* had no modifications in the digestive system, intestine and any missing extremities (antennae) or malformations (**Figure 5**). However, the mycelial ethyl acetate extract exposed to *A. nauplii* had noticeable changes in eye formation, eyeball shape, eyeball shrinking and weakened iris (**Figures 5A,B**). The intestinal swelling (**Figure 6a**) and malformations in the outer shell and antennae losses were observed (**Figure 6b**), while exposed to the mycelia extract.

**Table 2:** Biotoxicity of *P. daleae* mycelia ethyl acetate extract on *Artemia nauplii*.

Concentrations (μg/ml)	12 h			24 h		
	LC_50_ (LCL-UCL) (μg/ml)	LC_90_ (LCL-UCL) (μg/ml)	χ^2^ (*df* = 7)	LC_50_ (LCL-UCL) (μg/ml)	LC_90_ (LCL-UCL) (μg/ml)	χ^2^ (*df* = 7)
50						
200	2.167	5.295		0.290	0.609	
300	(0.056–8.504)	(0.293–15.803)	2.840	(0.04–1.775)	(0.013–3.002)	3.822

*Control (Salt water) – nil mortality. LC_*50*_, lethal concentration that kills 50% of the exposed larvae; LC_*90*_, lethal concentration that kills 90% of the exposed larvae; LCL, lower confidence limit; UCL, upper confidence limit; *df*, degree of freedom; χ^*2*^, chi-square values are significant at *P <* 0.05 level. Mean value of five replicates.*

## Discussion

Currently, there is an increased attention toward the use of natural insecticides to reduce the synthetic insecticides and their residues are a basis of environmentally associated hazards and insecticides resistance (Amer and Mehlhorn, 2006a). Hence, the screening of microbial products is potential insecticides to establish their potential as a feasible alternative for control of vector-borne diseases (Ignacimuthu and Paulraj, 2009). The major advantages for this method, they kill the larval instars and nil effects on non-target organisms and produce the stability of extracellular metabolites can be build a promising alternative fungal based biolarvicides (Vyas et al., 2007). The microbial insecticides are generally pest-specific, readily biodegradable and usually lack toxicity to higher animals (Dhanasekaran and Thangaraj, 2014). Examples from mycoinsecticides include species, *Metarhizium* and *Lecanicillium*, which can infect hundreds of insect hosts from different genera and showed a higher level of host specificity (Jeger et al., 2009).

Mohanty and Prakash (2000), Priyanka et al. (2001) have described that the *Trichophyton ajelloi* and *Chrysosporium tropicum* filtrate metabolites are effective on larvae of *Cx. quinquefasciatus* and *An. stephensi*. In addition, Vijayan and Balaraman (1991) have previously reported the metabolites of 17 fungi are effective larvicidal agent against the 3rd instar of *An. stephensi* and *Cx. quinquefasciatus*. The entomopathogenic fungi also produced extracellular metabolites to have become a focus of interest for insect pathologists (Kucera and Samsinakova, 1968). Few entomopathogenic fungi, *Paecilomyces fumosoroseus, Beauveria bassiana*, and *F. moniliforme* produce mosquito larvicidal compounds like cyclodepsipeptide, as well as beauvericin and enniatin (Vyas et al., 2006a).

In the present study, soil samples were collected from various localities of Karumandurai hills for isolation of filamentous fungus *Penicillium*. The taxonomic identification of *Penicillium* sp. had complex and reported as diverse number of species (nearly 250), with tiny variations (Patricia et al., 2007). Use an appropriate culture media and their morphological features were used for identification and this method was difficult to identifying all the species (Pitt, 1980, 1993, 1995; Frisvad, 1981, 1985). Recently, molecular marker methods for identification of fungi based on the variability of ribosomal genes by ITS and rDNA gene cluster (Gardes and Bruns, 1993). According to the analog result of the fungal sequence by BLAST on NCBI, all the obtained sequences shared 98% similarity to *Penicillium* sp..

In tropical countries, the fungal intra/extra metabolites have been proved to be powerful agent for controlling malaria and filariasis (Singh and Prakash, 2010). In the present study, isolated *P. daleae* mycelia ethyl acetate extract showed dose dependent larvicidal activity. The first 24 h treatment, less than 40% the doses showed mortality, while the 48-h exposure revealed that doses >500 μg/ml, are possible to kill >50% tested larvae populations. Earlier report stated that the toxicity of *F. oxysporum* against the larvae of *Cx. quinquefasciatus*, under *in vitro* (Demain and Fang, 2000; Prakash et al., 2010), resulted bioactive metabolites from *Fusarium* sp. (Czapek-Dox broth) shown most efficient against I–IV instars larvae of *An. stephensi*. Considerable observations were made in the understanding the role of fungal bioactive compounds for penetration of host cuticle and it produce mosquito larvicial toxins. Similarly, Trabousli et al. (2002) reported *Cx. pipiens* biotype *molestus* mortality shown concentration-dependent activity (1–2 days exposure).

After treatment of *P. daleae* metabolites, the larval survival rate of *Ae. aegypti* was considerably reduced with the following the LC_50_ values (105.077, 83.943, and 76.513; LC_90_ = 128.035, 106.869, 125.640, and 104.606 μg/ml). Similarly, Siddhardha et al. (2010) found *A. funiculosus* showed larvicidal toxicity against III–IV instar larvae of *Ae. aegypti* (LC_50_ = 204.51 and 271.64 ppm). More recently, several researchers are searching novel bio-based insecticides and insect growth regulators for control of mosquitoes (Melo-Santos et al., 2001; Sihunincha et al., 2005; Vythilingam et al., 2005; Wang and Jaal, 2005; Pontes et al., 2010). Earlier findings have reported the use of microorganisms like *Bacillus sphaericus* (Poopathi and Tyagi, 2002; Singh and Prakash, 2009). *A. terreus* (Ragavendran and Natarajan, 2015) Keratinophilic soil fungi (Vijayan and Balaraman, 1991; Mohanty and Prakash, 2000) *Tolypocladium cylindrosporum* and *Culicinomyces clavisporus* species (Serit and Yap, 1984; Seif and Shaarawi, 2003) having bioactive constituents for larvicidal and pupicidal effects against *An. stephensi, Cx. quinquefasciatus*, and *Ae. aegypti*. Similarly, Govindarajan et al. (2005), Thomas and Read (2007) identified potential biolarvicides against selected mosquitoes in the lab and field conditions.

The microscopic and histopathological analysis clearly indicates that mycelial ethyl acetate extract damaged the various parts of (head, thorax, midgut, and anal gill regions) third instar larvae of *Ae. aegypti* and *Cx. quinquefasciatus*. Previously, Vijayakumar et al. (2015) investigated that nano-based treatment direct to disorganized and disappeared epithelial layer in treated larvae of *Ae. aegypti* and affected tissues of antennae, midgut, hindgut, gill regions and nerve ganglia of larvae *An. stephensi* and *Cx. quinquefasciatus* treated with *P. amboinicus*. Recently, Suganya et al. (2017) studied histopathological effects against the Zika virus vector *Ae. aegypti* complete disintegration of abdominal region, particularly in the midgut and caeca, with loss of lateral and caudal hairs and stereomicroscopic observation on *Ae. aegypti*, shown losses of head hairs (upper and lower), antenna and caudal hairs.

In control *A. nauplii* show an average swimming speed in the testing wells. After 24 h treated with ethyl extract of *P. daleae* the swimming speed of organisms were motionless or inhibited. Similarly, Charoy et al. (1995), Charoy and Janssen (1999), Larsen et al. (2008) reported treatment of toxic compounds to *Artemia* sp. larvae observed on swimming speed alterations. *A. nauplii* exposure to *P. daleae* mycelium extract at lower concentration did not induce any significant lethal effects and increase concentration to *Artemia* sp. revealed marginal death, respectively. The healthy *Artemia* sp. individuals are active swimmers, thus, alterations in swimming activity are valid as behavioral endpoints to detect stress at sub-lethal concentrations of various toxic compounds (Garaventa et al., 2010). Recently, adult copepod *Tigriopus japonicus* exposed to *Fukuyoa* sp. showed a decrease in activity, loss of motor control, and abnormal swimming (Lee et al., 2014).

The biotoxicity of *P. daleae* ethyl acetate extract was evaluated on non-target organism *A. nauplii*. The results achieved negligible toxicity (two time intervals 12 and 24 h) on *A. nauplii*, with LC_50_ and LC_90_ values ranged from 2.167, 0.290 to 5.295, 0.609 μg/ml. Currently insignificant toxicity was noticed during the toxicity test of mosquitocidal reflect of particles to the non-target aquatic organisms (Benelli, 2016). Subramani et al. (2013) reported the *Penicillium* sp. metabolites exhibiting strong cytotoxicity against brine shrimp (LD_50_ of 96 μg/ml). Ding et al. (2008) studied the fungus *Cladosporium* sp. extracts tested against brine shrimp lethality test revealing no cytotoxicity, which indicates the ideal eco-friendly nature. Similarly, Barakat and Gohar (2012) observed notable toxicity on brine shrimp by ethyl acetate extract of marine *A. terreus* (LC_50_ = 32 μg/ml). The microbes from marine extracts caused significant lethality on *A. salina* (LC_50_ < 1,000 μg/ml) and the improved lethal activity based on time exposed (Ordaz et al., 2010). Overall, the findings of the present investigation report *P. daleae* exhibiting better larvicidal effects against two vital vector mosquitoes.

## Conclusion

The fungal derived products and their metabolites play a significant role in the development an efficient natural mosquito control drugs. For molecular identification of *Penicillium* sp., ITS markers were found as effective tool. In this present study, the isolates showed strong connection and consistency in morphological and molecular data. The findings suggests that mycelial ethyl acetate extract from *P. daleae* (KX387370) are potential natural mosquito larvicides. However, *P. daleae* metabolites exhibited 100% mortality of *Ae. aegypti* and *Cx. quinquefasciatus* (I–II instar larvae) at desired concentration (1000 μg/ml) of metabolites. Histopathological analysis highlighted fungal based metabolites-triggered tissue changes and losses of cuticular membrane. Our obtained results confirm that swimming behavior of *A. nauplii* were treated with *P. daleae* mycelium extract at various concentrations and observed marine larvae behavioral pattern to be applied in ecotoxicology. This result is very important in order to reduce the efforts in rearing test organisms. The acute exposure of *A. nauplii* larvae to these different concentrations of *P. daleae* ethyl acetate extract induces significant changes in their behavior, considerable mortality or oxidative stress within 12 h exposure. The continuous exposure (24 h) of extract induces an oxidative stress resulted with least mortality effects. Moreover, the outcome of study is providing strong scientific evidences for developing more selective, ideal and eco-friendly mosquito larvicidal agents.

## Ethics Statement

The national and international rules and regulations followed, while the use of animals/insects during the experimental purpose.

## Author Contributions

Construct the research plan: CR and DN Perform the experimental work, analysis the results and manuscript preparation: CR. Revision and grammatical correction of the manuscript: DN and TM. Finally, all authors have clearly read and approved the final manuscript.

## Conflict of Interest Statement

The authors declare that the research was conducted in the absence of any commercial or financial relationships that could be construed as a potential conflict of interest.

## Footnotes

1 www.ncbi.nlm.nih.gov/BLAST

2 http://www.ebi.ac.uk/Tools/clustalw2/index.html

## References

[B1] Abbott W. S. (1925). A method of computing the effectiveness of insecticides. *J. Econ. Entomol.* 18 265–267. 10.1093/jee/18.2.265a

[B2] Alyuruk H. Demir G. K. Cavas L. (2013). A video tracking based improvement of acute toxicity test on *Artemia salina*. *Mar. Freshw. Behav. Physiol.* 46 251–266. 10.1080/10236244.2013.814224

[B3] Amer A. Mehlhorn H. (2006a). Larvicidal effects of various essential oils against *Aedes, Anopheles*, and *Culex* larvae (*Diptera, Culicidae*). *Parasitol. Res.* 99 466–472. 16642386 10.1007/s00436-006-0182-3

[B4] Amer A. Mehlhorn H. (2006b). Repellency effect of forty-one essential oils against *Aedes, Anopheles* and *Culex* mosquitoes. *Parasitol. Res.* 99 478–490. 16642384 10.1007/s00436-006-0184-1

[B5] Banumathi B. Vaseeharan B. Chinnasamy T. Vijayakumar S. Govindarajan M. Naiyf S. A. (2017). *Euphorbia rothiana*-fabricated Ag nanoparticles showed high toxicity on *Aedes aegypti* larvae and growth inhibition on microbial pathogens: a focus on morphological changes in Mosquitoes and Antibiofilm potential against Bacteria. *J. Clust. Sci.* 28 2857–2872. 10.1007/s10876-017-1263-4

[B6] Barakat K. M. Gohar Y. M. (2012). Antimicrobial agents produced by marine *Aspergillus terreus* var. africanus against some virulent fish pathogens. *Ind. J. Microbiol.* 52 366–372. 10.1007/s12088-012-0255-1 23997326 PMC3460127

[B7] Barron G. L. (1968). *The Genera of Hyphomycetes from the Soil.* Baltimore, MD: The Williams & Wilkins Co 364.

[B8] Belofsky G. N. Jensen P. R. Renner M. K. Fenical W. (1998). New cytotoxic sesquiterpenoid nitrobenzoyl esters from a marine isolate of the fungus *Aspergillus versicolor*. *Tetrahedron* 54 1715–1724. 10.1016/S0040-4020(97)10396-9

[B9] Benelli G. (2015a). Research in mosquito control: current challenges for a brighter future. *Parasitol. Res.* 114 2801–2805. 10.1007/s00436-015-4586-9 26093499

[B10] Benelli G. (2015b). Plant-borne ovicides in the fight against mosquito vectors of medical and veterinary importance: a systematic review. *Parasitol. Res.* 114 3201–3212. 10.1007/s00436-015-4656-z 26239801

[B11] Benelli G. (2016). Plant-mediated biosynthesis of nanoparticles as an emerging tool against mosquitoes of medical and veterinary importance: a review. *Parasitol. Res.* 115 23–24. 10.1007/s00436-015-4800-9 26541154

[B12] Benelli G. Flamini G. Canale A. Cioni P. L. Conti B. (2012a). Toxicity evaluation of different essential oil formulations against the Mediterranean Fruit Fly *Ceratitis capitata* (Wiedemann) (Diptera Tephritidae). *Crop Prot.* 42 223–229. 10.1016/j.cropro.2012.05.024

[B13] Benelli G. Flamini G. Canale A. Molfetta I. Cioni P. L. Conti B. (2012b). Repellence of *Hyptis suaveolens* whole essential oil and major constituents against adults of the granary weevil *Sitophilus granarius*. *Bull. Insectol.* 65 177–183.

[B14] Benserradj O. Mihoubi I. (2014). Larvicidal activity of entomopathogenic fungi *Metarhizium anisopliae* against mosquito larvae in Algeria. *Int. J. Curr. Microbiol. Appl. Sci.* 3 54–62.

[B15] Berdy J. (2005). Bioactive microbial metabolites. *J. Antibiot.* 58 1–26. 10.1038/ja.2005.1 15813176

[B16] Burnett J. H. (1975). *Mycogenetics.* London: John Wiley and Sons 375.

[B17] Charoy C. Janssen C. R. (1999). The swimming behaviour of *Brachionus calyciflorus* (rotifer) under toxic stress. II. Comparative sensitivity of various behavioural criteria. *Chemosphere* 38 3247–3260. 10.1016/j.chemosphere.2013.08.086 24079998

[B18] Charoy C. P. Janssen C. R. Persoone G. Clement P. (1995). The swimming behaviour of *Brachionus calyciflorus* (rotifer) under toxic stress. I. The use of automated trajectory for determining sublethal effects of chemicals. *Aquat. Toxicol.* 32 271–282. 10.1016/j.chemosphere.2013.08.086 24079998

[B19] Choi Y. W. Hyde K. D. Ho W. H. (1999). Single spore isolation of fungi. *Fungal Divers.* 3 29–38.

[B20] Conti B. Benelli G. Flamini G. Cioni P. L. Profeti R. Ceccarini L. (2012). Larvicidal and repellent activity of *Hyptis suaveolens* (Lamiaceae) essential oil against the mosquito *Aedes albopictus* Skuse (Diptera: Culicidae). *Parasitol. Res.* 110 2013–2021. 10.1007/s00436-011-2730-8 22160253

[B21] Crowley N. Bradley J. M. Darrell J. H. (1969). *Practical Bacteriology.* London: Butterworth and Co. 164–168.

[B22] Demain A. L. Fang A. (2000). “The natural functions of secondary metabolites in history of modern biotechnology,” in *Advances in Biochemical Engineering Biotechnology* Vol. 69 ed. Scheper T. (Berlin: Springer) 1–39. 10.1007/3-540-44964-7_1 11036689

[B23] Dhanasekaran D. Thangaraj R. (2014). Microbial secondary metabolites are an alternative approaches against insect vector to prevent zoonotic diseases. *Asian Pac. J. Trop. Dis.* 4 253–261. 10.1016/S2222-1808(14)60569-7

[B24] Ding L. Qin S. Li F. Chi X. Laatsch H. (2008). Isolation, antimicrobial activity, and metabolites of fungus *Cladosporium* sp. associated with Red Alga *Porphyra yezoensis*. *Curr. Microbiol.* 56 229–235. 10.1007/s00284-007-9063-y 18214603

[B25] Domsch K. H. Gams W. Anderson T. H. (1980). *Compendium of Soil Fungi.* London: Academic Press 672.

[B26] Doyle J. J. (1990). Isolation of plant DNA from fresh tissue. *Focus* 12 13–15.

[B27] Finney D. J. (1971). *Probit Analysis.* London: Cambridge University Press 68–78.

[B28] Frisvad J. C. (1981). Physiological criteria and mycotoxin production as aids in identification of common asymmetric penicillia. *Appl. Environ. Microbiol.* 41 568–579. 16345727 10.1128/aem.41.3.568-579.1981 PMC243741

[B29] Frisvad J. C. (1985). Creatine sucrose agar, a differential medium for mycotoxin producing terveticillate *Penicillium* species. *Lett. Appl. Microbiol.* 1 109–113. 10.1111/j.1472-765X.1985.tb01500.x

[B30] Garaventa F. Gambardella C. Di Fino A. Pittore M. Faimali M. (2010). Swimming speed alteration of *Artemia* sp. and *Brachionus plicatilis* as a sub-lethal behavioural endpoint for ecotoxicological surveys. *Ecotoxicology* 19 512–519. 10.1007/s10646-010-0461-8 20099027

[B31] Gardes M. Bruns T. D. (1993). ITS primers with enhanced specificity ors basidiomycetes – application to the identification of mycorrhizae and rust. *Mol. Ecol.* 2 113–118. 10.1111/j.1365-294X.1993.tb00005.x 8180733

[B32] Govindarajan M. Jebanesan A. Reetha D. (2005). Larvicidal effect of extracellular secondary metabolites of different fungi against the mosquito, *Culex quinquefasciatus* Say. *Trop. Biomed.* 22 1–3. 16880747

[B33] Govindarajan M. Mathivanan T. Elumalai K. Krishnappa K. Anandan A. (2011). Mosquito larvicidal, ovicidal, and repellent properties of botanical extracts against *Anopheles stephensi, Aedes aegypti*, and *Culex quinquefasciatus* (Diptera: Culicidae). *Parasitol. Res.* 109 353–367. 10.1007/s00436-011-2263-1 21318385

[B34] Govindarajan M. Sivakumar R. (2012). Adulticidal and repellent properties of indigenous plant extracts against *Culex quinquefasciatus* and *Aedes aegypti* (Diptera: Culicidae). *Parasitol. Res.* 110 1607–1620. 10.1007/s00436-011-2669-9 22009267

[B35] Hall T. A. (1999). BioEdit: a user-friendly biological sequence alignment editor and analysis program for Windows 95/98/NT. *Nucleic Acids Symp. Ser.* 41 95–98.

[B36] Hamill R. L. Higgens C. Boaz Z. E. (1969). The structure of beauvericin, a new depsipeptide antibiotic toxic to *Artemia* salvia. *Tetrahedron Lett.* 49 4255–4258. 10.1016/S0040-4039(01)88668-8

[B37] Holler U. (1999). *Isolation, Biological Activity and Secondary Metabolite Investigations of Marine-derived Fungi and Selected Host Sponges.* Ph.D. thesis, Technische Universität Braunschweig Braunschweig.

[B38] Ignacimuthu S. Paulraj M. G. (2009). Non-chemical insect pest management. *Curr. Sci.* 97 136–137.

[B39] James A. A. (1992). Mosquito molecular genetics: the hands that feed bite back. *Science* 257 37–38. 10.1126/science.1352413 1352413

[B40] Jeger M. J. Jeffries P. Elad Y. Xu X. M. (2009). A generic theoretical model for biological control of foliar plant diseases. *J. Theor. Biol.* 256 201–214. 10.1016/j.jtbi.2008.09.036 18983855

[B41] Kamel E. A. Rashed M. E. (2014). Electrophoretic protein banding patterns among *Penicillium* strains isolated from Saudi Arabia. *Int. J. Appl. Sci. Biotechnol.* 2 283–290. 10.3126/ijasbt.v2i3.10949

[B42] Klich M. A. Pitt J. I. (1992). *A Laboratory Guide to the Common Aspergillus Species and their Teleomorphs.* North Ryde, NSW: Commonwealth Scientific and Industrial Research Organisation 116.

[B43] Kornerup A. Wanscher J. H. (1967). *Methuen Handbook of Colour* 2nd Edn. London: Methuen 243.

[B44] Kucera M. Samsinakova A. (1968). Toxins of the entomophagous fungus *Beauveria bassiana*. *J. Invertebr. Pathol.* 12 316–320. 10.1016/0022-2011(68)90333-9 5752486

[B45] Lam Y. K. T. Dai P. Borris R. Dombrowski A. Ransom R. Young G. (1994). A new indole from *Penicillium daleae*. *J. Antibiot.* 47 724. 10.7164/antibiotics.47.724 8040078

[B46] Larsen P. S. Madsen C. V. Riisgard H. U. (2008). Effect of temperature and viscosity on swimming velocity of the copepod *Acartia tonsa*, brine shrimp *Artemia salina* and rotifer *Brachionus plicatilis*. *Aquat. Biol.* 4 47–54. 10.3354/ab00093

[B47] Lee K. W. Kang J. H. Baek S. H. Choi Y. U. Lee D. W. Park H. S. (2014). Toxicity of the dinoflagellate *Gambierdiscus* sp. toward the marine copepod *Tigriopus japonicus*. *Harmful Algae* 37 62–67. 10.1016/j.hal.2014.05.007

[B48] Lin Y. Shao Z. Jiang G. Zhou S. Cai J. Vrijmoed L. L. P. (2000). Penicillazine, a unique quinolone derivative with 4H-5 6-Dihydro-12- oxazine ring system from the marine fungus *Penicillium sp*. (strain #386) from the South China Sea. *Tetrahedron* 56 9607–9609. 10.1016/S0040-4020(00)00917-0

[B49] Matha V. Weiser J. A. Olejricek R. (1988). The effect of Tolypin on *Tolypocladium niveum* crude extract against mosquito and black fly larvae in laboratory. *Folia Parasitol.* 35 379–381. 2906895

[B50] Maurya P. Mohan L. Sharma P. Srivastava C. N. (2011). Evaluation of larvicidal potential of certain insect pathogenic fungi extracts against *Anopheles stephensi* and *Culex quinquefasciatus*. *Entomol. Res.* 41 211–215. 10.1111/j.1748-5967.2011.00347.x

[B51] Mehmet A. Demir V. Arslan Z. Camas M. Celik F. (2016). Toxicity of engineered nickel oxide and cobalt oxide nanoparticles to *Artemia salina* in seawater. *Water Air Soil Pollut.* 227 70. 27152058 10.1007/s11270-016-2771-9 PMC4852876

[B52] Melo-Santos M. A. Sanches E. G. de Jesus F. J. Regis L. (2001). Evaluation of a new tablet formulation based on *Bacillus thuringiensis* sorovar *israelensis* for larvicidal control of *Aedes aegypti*. *Mem. Inst. Oswaldo Cruz* 96 859–860. 10.1590/S0074-02762001000600020 11562715

[B53] Misato T. (1983). “Recent status and future aspects of agricultural antibiotics,” in *Natural Products Pesticide Chemistry: Human Welfare and the Environment* Vol. 2 eds Miyamoto J. Kearney P. C. (Oxford: Pergamon Press) 241–246.

[B54] Mohanty S. S. Prakash S. (2000). Laboratory evaluation of *Trichophyton ajelloi*, a fungal pathogen of *Anopheles stephensi* and *Culex quinquefasciatus*. *J. Am. Mosq. Control Assoc.* 16 254–257. 11081656

[B55] OECD (2004). *Guideline for Testing of Chemicals—Daphnia sp. Acute Immobilisation Test.* Paris: OECD 202.

[B56] Ordaz G. D’Armas H. Yanez D. Hernandez J. Camacho A. (2010). Secondary metabolites, lethality and antimicrobial activity of extracts from three corals and three marine mollusks from Sucre, Venezuela. *Rev. Biol. Trop.* 58 677–688. 20527468

[B57] Patricia G. C. de Queiroz M. V. Pereira O. L. de Araujo E. F. (2007). Morphological and molecular differentiation of the pectinase producing fungi *Penicillium expansum* and *Penicillium griseoroseum*. *Braz. J. Microbiol.* 38 71–77. 10.1590/S1517-83822007000100015

[B58] Pavela R. (2015a). Essential oils for the development of eco-friendly mosquito larvicides: a review. *Ind. Crops Prod.* 76 174–187. 10.1016/j.indcrop.2015.06.050

[B59] Pavela R. (2015b). Acute toxicity and synergistic and antagonistic effects of the aromatic compounds of some essential oils against *Culex quinquefasciatus* Say larvae. *Parasitol. Res.* 114 3835–3853. 10.1007/s00436-015-4614-9 26149532

[B60] Persoone G. Van de Vell A. Van Steertegem M. Nayer B. (1989). Predictive value for laboratory tests with aquatic invertebrates: influence of experimental conditions. *Aquat. Toxicol.* 14 149–166. 10.1016/0166-445X(89)90025-8

[B61] Persoone G. Wells P. G. (1987). “*Artemia* in aquatic toxicology: a review,” in *Artemia Research and its Application (Morphology, Genetics, Strain Characterization, Toxicology* Vol. 1 eds Sorgeloos P. Bengtson D. A. Decleir W. Jasper F. (Wetteren: Universa Press) 259–275.

[B62] Peter H. Matha V. Roberts D. W. (1989). “Enzymes involved in the synthesis of fungal toxins,” in *Proceeding of the International Conference Biopesticide, Theory and Practice* české Budějovice 169–181.

[B63] Phara K. D. Kommedahl T. A. (1954). Modified plating technique for the study of soil fungi. *Phytopathology* 44 502.

[B64] Pitt J. I. (1979). *The Genus Penicillium and its Teleomorphic States Eupenicillium and Talaromyces.* London: Academic Press 629.

[B65] Pitt J. I. (1980). *The Genus Penicillium and its Teleomorphic States Eupenicillium and Talaromyces.* London: Academic Press.

[B66] Pitt J. I. (1993). “Speciation and evolution in *Penicillium* and related genera,” in *The Fungal Holomorph: Mitotic, Meiotic and Pleomorphic Speciation in Fungal Systematics* eds Reynolds D. R. Taylor W. (Wallingford: CAB International) 113–117.

[B67] Pitt J. I. (1995). Phylogeny in the genus *Penicillium*: a morphologist’s perspective. *Can. J. Bot.* 73 768–777. 10.1139/b95-321

[B68] Pontes R. J. S. Filho F. F. D. de Alencar C. H. M. Regazzi A. C. F. Cavalcanti L. P. G. Ramos A. N. Jr. (2010). Impact of water renewal on the residual effect of larvicides in the control of *Aedes aegypti*. *Mem. Inst. Oswaldo Cruz* 105 220–224. 10.1590/S0074-02762010000200019 20428685

[B69] Poopathi S. Tyagi B. K. (2002). Studies on *Bacillus sphaericus* toxicity related resistance development and biology in the filariasis vector *Culex quinquefasciatus* (Diptera:Culicidae) from South India. *Appl. Entomol. Zool.* 37 365–371. 10.1303/aez.2002.365

[B70] Prakash S. Singh G. Soni N. Sharma S. (2010). Pathogenicity of *Fusarium oxysporum* against the larvae of *Culex quinquefasciatus* (Say) and *Anopheles stephensi* (Liston) in laboratory. *Parasitol. Res.* 107 651–655. 10.1007/s00436-010-1911-1 20499096

[B71] Priyanka S. Prakash S. (2003). Laboratory efficacy test for fungal metabolites of *Chrysosporium tropicum* against *Culex quinquefasciatus*. *J. Am. Mosq. Control Assoc.* 19 404–407. 14710744

[B72] Priyanka S. Srivastava J. N. Prakash S. (2001). *Chrysosporium tropicum* efficacy against *Anopheles stephensi* larvae in the laboratory. *J. Am. Mosq. Control Assoc.* 17 127–130. 11480820

[B73] Radhika Rajasree S. R. Ganesh Kumar V. Stanley Abraham L. Indabakandan D. (2010). Studies on the toxicological effects of engineered nanoparticles in environment—a review. *Int. J. Appl. Bioeng.* 4 44–53. 10.18000/ijabeg.10070

[B74] Ragavendran C. Natarajan D. (2015). Insecticidal potency of *Aspergillus terreus* against larvae and pupae of three mosquito species *Anopheles stephensi, Culex quinquefasciatus*, and *Aedes aegypti*. *Environ. Sci. Pollut. Res.* 22 17224–17237. 10.1007/s11356-015-4961-1 26139412

[B75] Rambaut A. (2009). *FigTree. Tree Figure Drawing Tool Version 1.3.1.* Edinburgh: University of Edinburgh.

[B76] Raper K. B. Thom C. (1949). *A Manual of the Penicillia.* Baltimore, MD: Williams & Wilkins Co 875.

[B77] Regnault-Roger C. Vincent C. Arnason J. T. (2012). Essential oils in insect control: low-risk products in a high-stakes world. *Annu. Rev. Entomol.* 57 405–424. 10.1146/annurev-ento-120710-100554 21942843

[B78] Roberts D. W. (1966). Toxins from entomogenous fungus *Metarhizium anisopliae*. II. Symptoms and detection in moribund hosts. *J. Invert. Pathol.* 8 222–227. 10.1016/0022-2011(66)90132-7 5949416

[B79] Roberts D. W. (1967). “Some effects of *Metarhizium anisopliae* and its toxins on mosquito larvae,” in *Insect Pathol and Microbial Entomol* ed. Van der laan P. A. (Amsterdam: North-Holland Publishing Company) 243–246.

[B80] Rolhf F. J. (1990). *NTSYSPc. Numerical Taxonomy and Multivariant Analysis System. Version 2.02.* New York, NY: Applied Biostatistics.

[B81] Saitou N. Nei M. (1987). The neighbor-joining method: a new method for reconstructing phylogenetic trees. *Mol. Biol. Evol.* 4 406–425. 3447015 10.1093/oxfordjournals.molbev.a040454

[B82] Seif A. I. Shaarawi F. A. (2003). Preliminary field trials with *Culicinomyces clavosporus* against some Egyptian mosquitoes in selected habitats. *J. Egypt. Soc. Parasitol.* 33 291–304. 12739818

[B83] Senthil-Nathan S. Savitha G. George D. K. Narmadha A. Suganya L. Chung P. G. (2006). Efficacy of *Melia azedarach* L. extract on the malarial vector *Anopheles stephensi* Liston. *Bioresour. Technol.* 97 1214–1221. 10.1016/j.biortech.2005.09.034 16054356

[B84] Serit M. A. Yap H. H. (1984). Comparative bioassays of *Tolypocladium cylindrosporum* gams (Californian strain) against four species of mosquitoes in Malaysia. *Southeast Asian J. Trop. Med. Public Health* 15 331–336. 6151744

[B85] Severini C. Rom R. Marinucci M. Rajmond M. (1993). Mechanisms of insecticide resistance in field populations of *Culex pipiens* from Italy. *J. Am. Mosq. Control Assoc.* 9 164–168. 7688795

[B86] Siddhardha B. Murty U. S. N. Narasimhulu M. Venkateswarlu Y. (2010). Isolation, characterization and biological evaluation of secondary metabolite from *Aspergillus funiculosus*. *Ind. J. Microbiol.* 50 225–228. 10.1007/s12088-010-0044-7 23100833 PMC3450324

[B87] Sihunincha M. Zamora-Perea E. Orellana-Rios W. Stancil J. D. Lopez-Sifuentes V. Vidal-Ore C. (2005). Potential use of pyriproxyfen for control of *Aedes aegypti* (Diptera: Culicidae) in Iquitos. *Peru. J. Med. Entomol.* 42 620–630 10.1093/jmedent/42.4.620 16119551

[B88] Singh G. Prakash S. (2009). Efficacy of *Bacillus sphaericus* against larvae of malaria and filarial vectors: an analysis of early resistance detection. *Parasitol. Res.* 104 763–766. 10.1007/s00436-008-1252-5 18989699

[B89] Singh G. Prakash S. (2010). Fungi *Beauveria bassiana* (Balsamo) metabolites for controlling malaria and filaria in tropical countries. *Adv. Biomed. Res.* 21 238–242.

[B90] Sivagnaname N. Kalyanasundaram M. (2004). Laboratory evaluation of methanolic extract of *Atlantia monophylla* (Family: Rutaceae) against immature stages of mosquitoes and non-target Organisms. *Mem. Inst. Oswaldo Cruz* 99 115–118. 10.1590/S0074-02762004000100021 15057359

[B91] Soni N. Prakash S. (2010). Effect of *Chrysosporium keratinophilum* metabolites against *Culex quinquefasciatus* after chromatographic purification. *Parasitol. Res.* 107 1329–1336. 10.1007/s00436-010-2003-y 20689969

[B92] Sorgeloos P. (1980). Availability of reference *Artemia* cysts. *Mar. Ecol. Prog. Ser.* 3 363–364. 10.3791/3790 22525984 PMC3598398

[B93] Sorgeloos P. Van DerWielen C. R. Persoone G. (1978). The use of *Artemia nauplii* for toxicity tests—a critical analysis. *Ecotoxicol. Environ. Saf.* 2 249–255. 10.1016/S0147-6513(78)80003-7 751788

[B94] Subramani R. Kumar R. Prasad P. Aalbersberg W. (2013). Cytotoxic and antibacterial substances against multi-drug resistant pathogens from marine sponge symbiont: citrinin, a secondary metabolite of *Penicillium* sp. *Asian Pac. J. Trop. Biomed.* 3 291–296. 10.1016/S2221-1691(13)60065-9 23620853 PMC3634926

[B95] Suganya P. Vaseeharan B. Vijayakumar S. Balan B. Govindarajan M. Alharbi N. S. (2017). Biopolymer zein-coated gold nanoparticles: synthesis, antibacterial potential, toxicity and histopathological effects against the Zika virus vector *Aedes aegypti*. *J. Photochem. Photobiol. B Biol.* 173 404–411. 10.1016/j.jphotobiol.2017.06.004 28654862

[B96] Tamura K. Nei M. Kumar S. (2004). Prospects for inferring very large phylogenies by using the neighbor-joining method. *Proc. Natl. Acad. Sci. U.S.A.* 101 11030–11035. 10.1073/pnas.0404206101 15258291 PMC491989

[B97] Tamura K. Peterson D. Peterson N. Stecher G. Nei M. Kumar S. (2011). MEGA5: molecular evolutionary genetics analysis using maximum likelihood, evolutionary distance, and maximum parsimony methods. *Mol. Biol. Evol.* 10 2731–2739. 10.1093/molbev/msr121 21546353 PMC3203626

[B98] Thomas M. B. Read A. F. (2007). Can fungal biopesticides control malaria? *Nat. Microbiol. Rev.* 5 377–383. 10.1038/nrmicro1638 17426726

[B99] Tiwari K. L. Jadhav S. K. Kumar A. (2011). Morphological and molecular study of different *Penicillium* species. *Middle East J. Sci. Res.* 7 203–210.

[B100] Trabousli A. F. Taoubi K. Samih E. H. Bessiere J. M. Rammal S. (2002). Insecticidal properties of essential oils against the mosquito *Culex pipiens molestus* (Diptera: Culicidae). *Pest Manag. Sci.* 58 491–495. 10.1002/ps.486 11997977

[B101] Tuite J. (1961). Fungi isolated from unstored corn seed in Indian in 1956 - 1988. *Plants Dis. Rep.* 45 212–215.

[B102] Vasilakis N. Shell E. J. Fokam E. B. Mason P. W. Hanley K. A. Estes D. M. (2007). Potential of ancestral sylvatic dengue-2 viruses to re-emerge. *Virol. J.* 58 402–412. 10.1016/j.virol.2006.08.049 17014880 PMC3608925

[B103] Venkateswara Rao J. Kavitha P. Jakka N. M. Sridhar V. Usman P. K. (2007). Toxicity of organophosphates on morphology and locomotor behavior in brine shrimp, *Artemia salina*. *Arch. Environ. Contam. Toxicol.* 53 227–232. 10.1007/s00244-006-0226-9 17549541

[B104] Vijayakumar S. Vinoj G. Malaikozhundan B. Shanthi S. Vaseeharan B. (2015). *Plectranthus amboinicus* leaf extract mediated synthesis of zinc oxide nanoparticles and its control of methicillin resistant *Staphylococcus aureus* biofilm and blood sucking mosquito larvae. *Spectrochim. Acta A Mol. Biomol. Spectrosc.* 137 886–891. 10.1016/j.saa.2014.08.064 25280336

[B105] Vijayan V. Balaraman K. (1991). Metabolites of fungi and actinobacteria active against mosquito larvae. *Ind. J. Med. Res.* 93 115–117. 1677347

[B106] Vyas N. Dua K. K. Prakash S. (2006a). Bioassay of secondary metabolites of *Lagenidium giganteum* on mosquito larvae for vector control. *Bull. Biol. Sci.* 4 65–69.

[B107] Vyas N. Dua K. K. Prakash S. (2006b). Laboratory efficacy of metabolites of *Lagenidium giganteum* (Couch) on *Anopheles stephensi* (Liston) after filtration by column chromatography. *J. Commun. Dis.* 38 176–180. 17370682

[B108] Vyas N. Dua K. K. Prakash S. (2007). Efficacy of *Lagenidium giganteum* metabolites on mosquito larvae with reference to nontarget organisms. *Parasitol. Res.* 101 385–390. 10.1007/s00436-007-0496-9 17334944

[B109] Vythilingam I. Maria Luz B. Hanni R. Siew Beng T. Cheong Huat T. (2005). Laboratory and field evaluation of the insect growth regulator pyriproxyfen (Sumilarv 0.5 G) against dengue vectors. *J. Am. Mosq. Control Assoc.* 21 296–300. 10.2987/8756-971X(2005)21[296:LAFEOT]2.0.CO;2 16252520

[B110] Wang L. Y. Jaal Z. (2005). Sub-lethal effects of *Bacillus thuringiensis* H-14 on the survival rate, longevity, fecundity and F1 generation developmental period of *Aedes aegypti*. *Dengue Bull.* 29 192–196.

[B111] Warcup J. H. (1950). The soil-plate method for isolation of fungi from soil. *Nature* 166 117–118. 10.1038/166117b0 15439172

[B112] Warcup J. H. (1955). Isolation of fungi from hyphae present in soil. *Nature* 175 953–954. 10.1038/175953b0 14383782

[B113] Warikoo R. Wahab N. Kumar S. (2011). Oviposition-altering and ovicidal potentials of five essential oils against female adults of the dengue vector. *Aedes aegypti* L. *Parasitol. Res.* 109 1125–1131. 10.1007/s00436-011-2355-y 21445613

[B114] Weber C. I. (1993). *Methods for Measuring the Acute Toxicity of Effluents and Receiving Waters to Freshwater and Marine Organisms* 4th Edn. Cincinnati, OH: Environmental Monitoring Systems Laboratory 167.

[B115] White T. J. Bruns T. Lee S. Taylor J. (1990). “Amplification and direct sequencing of fungal ribosomal RNA genes for phylogenetics,” in *PCR Protocols: A Guide to Methods and Applications* eds Innis A. Gelfand D. H. Sninsky J. J. (San Diego, CA: Academic Press) 315–322.

[B116] WHO (2013). *World Filariasis Report.* Geneva: Switzerland 282.

[B117] WHO (2015). *Dengue and Severe Dengue Fact Sheet. World Malaria Report.* Washington, DC: National Press Club 1–5.

[B118] Woese C. R. Stackebrandt E. Macke T. J. Fox G. E. (1985). A phylogenetic definition of the major eubacterial taxa. *Syst. Appl. Microbiol.* 6 143–151. 10.1016/S0723-2020(85)80047-3 11542017

[B119] World Health Organization (2005). *Report of the Eighth WHOPES Working Group Meeting.* Geneva: WHO.

[B120] Zaleski K. M. (1927). *Penicillium daleae. Bull. Int. Acad. Pol. Sci. Lett. Sér. B* 495–498.

